# Russian gonococcal antimicrobial susceptibility programme (RU-GASP) – resistance in *Neisseria gonorrhoeae* during 2009–2012 and NG-MAST genotypes in 2011 and 2012

**DOI:** 10.1186/1471-2334-14-342

**Published:** 2014-06-19

**Authors:** Anna Kubanova, Alexey Kubanov, Nataliya Frigo, Viktoria Solomka, Vera Semina, Denis Vorobyev, Rafil Khairullin, Magnus Unemo

**Affiliations:** 1 Тhe State Research Center of Dermatology, Venereology and Сosmetology of The Russian Ministry of Health (SRCDVC), Moscow, Russia; 2 WHO Collaborating Centre for Gonorrhoea and Other STIs, National Reference Laboratory for Pathogenic Neisseria, Department of Laboratory Medicine, Microbiology, Örebro University Hospital, SE-701 85 Örebro, Sweden

**Keywords:** *Neisseria gonorrhoeae*, Gonorrhoea, Antimicrobial resistance, National surveillance, Russian gonococcal antimicrobial susceptibility programme (RU-GASP), Extended-spectrum cephalosporins (ESCs), Ceftriaxone, Treatment, *N. gonorrhoeae* multiantigen sequence typing (NG-MAST), Russia

## Abstract

**Background:**

Antimicrobial resistance (AMR) in *Neisseria gonorrhoeae* is a major concern worldwide and gonococcal AMR surveillance globally is imperative for public health purposes. In Eastern Europe, gonococcal AMR surveillance is exceedingly rare. However, in 2004 the Russian gonococcal antimicrobial susceptibility programme (RU-GASP) was initiated. The aims of this study were to describe the prevalence and trends of gonococcal AMR from 2009 to 2012, and molecular epidemiological genotypes in 2011 and 2012 in Russia.

**Methods:**

Gonococcal isolates from 12–46 surveillance sites distributed across Russia, obtained in 2009 (n = 1200), 2010 (n = 407), 2011 (n = 423), and 2012 (n = 106), were examined for antimicrobial susceptibility using agar dilution method. Gonococcal isolates from 2011 and 2012 were investigated with *N. gonorrhoeae* multi-antigen sequence typing (NG-MAST).

**Results:**

During 2009–2012, the proportions of gonococcal isolates resistant to ciprofloxacin, penicillin G, azithromycin and spectinomycin ranged from 25.5% to 44.4%, 9.6% to 13.2%, 2.3% to 17.0% and 0.9% to 11.6%, respectively. Overall, the resistance level to penicillin G was stable, the resistance level to ciprofloxacin was decreasing, however, the level of resistance to azithromycin increased. All isolates were susceptible to ceftriaxone using the US CLSI breakpoints. However, using the European breakpoints 58 (2.7%) of the isolates were resistant to ceftriaxone. Interestingly, this proportion was decreasing, i.e. from 4.8% in 2009 to 0% in 2012.

**Conclusions:**

In Russia, the diversified gonococcal population showed a high resistance to ciprofloxacin, penicillin G and azithromycin. In general, the MICs of ceftriaxone were relatively high, however, they were decreasing from 2009 to 2012. Ceftriaxone should be the first-line for empiric antimicrobial monotherapy of gonorrhoea in Russia. It is essential to further strengthen the surveillance of gonococcal AMR (ideally also gonorrhoea treatment failures) in Russia.

## Background

Gonorrhoea is a major public health concern globally [1]. In 2011, 39 179 gonorrhoea cases were reported from 28 European Union (EU)/European Economic Area (EEA) Member States, with an overall incidence of 12.6 cases per 100,000 population [2]. In most of the non-EU/EEA countries of the World Health Organization (WHO) European Region (mostly in the former Soviet Union and Yugoslav Republic), during the last two decades the incidence has rapidly declined. This decline has also been observed in the largest non-EU/EEA country, that is, Russia (>142 million inhabitants). However, in 2011 Russia still reported an incidence of 38 cases per 100,000 population, which was the highest country incidence in the WHO European Region [2,3,http://data.euro.who.int/cisid]. Furthermore, the reported gonorrhoea incidences in Russia are underestimated, which is due to the large heterogeneity in health care settings nationally, differences in access to testing, suboptimal diagnostics, case reporting, e.g. lack of reporting of cases diagnosed in the private sector, and surveillance [3-5].

Unfortunately, the etiological agent of gonorrhoea, *Neisseria gonorrhoeae,* has developed antimicrobial resistance (AMR) to essentially all antimicrobials introduced as first-line treatment. Currently, ceftriaxone is the only recommended first-line option for antimicrobial monotherapy in many countries globally [6-12]. Most worryingly, rare verified treatment failure of pharyngeal gonorrhoea with ceftriaxone have been reported [13-18] and the first few extensively-drug resistant (XDR) gonococcal strains with high-level resistance to ceftriaxone were described recently [16,19,20]. In this era of hard-to-treat and possibly emergence of untreatable gonorrhoea, the WHO [21], European Centre for Disease Prevention and Control (ECDC) [22] and Centers for Disease Control and Prevention (CDC), USA [23] have published action/response plans to mitigate and control the spread of multidrug-resistant gonorrhoea. One key action emphasized for public health purposes in all these action/response plans is to substantially enhance the quality assured surveillance of gonococcal AMR worldwide [21-23].

In the WHO European Region, the European Gonococcal Antimicrobial Surveillance Programme (Euro-GASP) is operating in the EU/EEA since 2004. In Euro-GASP, 68% (21/31) of the EU/EEA countries are included in the gonococcal AMR surveillance [3,12]. However, in the non-EU/EEA countries of the WHO European Region, quality assured gonococcal AMR surveillance only exist in 13% (3/23) of the countries [3] and, in general, the awareness and knowledge regarding gonococcal AMR, which is crucial for informing the empirical treatment guidelines, is limited [3-5]. Nevertheless, in 2004 the national Russian GASP (RU-GASP) was initiated. The RU-GASP has been quality assured in accordance with WHO standards and, for international comparability of AMR data, the 2008 WHO *N. gonorrhoeae* reference strains are used as quality controls [24-27]. For molecular epidemiological typing of gonococci, the *N. gonorrhoeae* multiantigen sequence typing (NG-MAST) has been used in many countries worldwide [28]. However, very limited genetic characteristics of gonococcal strains circulating in Russia have been published and only two NG-MAST studies have been performed, examining isolates from 2004–2005 [29,30].

The aims of the present study were to examine the prevalence and trends of *N. gonorrhoeae* resistance, to previous and current antimicrobial treatment options, from 2009 to 2012 in Russia and the genotypic distribution of *N. gonorrhoeae*, by means of NG-MAST, isolated in 2011 and 2012 in Russia.

## Methods

### Study population

As previously described [24,25], dermatovenereological dispensaries situated all over Russia are surveyed in RU-GASP. In the present study, mainly consecutive culture positive gonorrhoea patients attending 12–46 dispensaries from January 2009 to December 2012 were included. Urethral and cervical specimens from females and urethral specimens from males were collected. All specimens were cultured on selective gonococcal agar media, and the *N. gonorrhoeae* isolates were preserved in cryomedium at -70°C and subsequently transported to the SRCDV for complete species verification and centralized AMR testing, as previously described [24,25]. At the SRDCDV, all isolates were confirmed as *N. gonorrhoeae* by identification of typical colonies on selective culture agar media, Gram negative diplococci in microscopy, rapid oxidase reaction, and a sugar utilization test [31]. All examined gonococcal isolates were cultured and stored as part of the routine diagnostics (standard care) and no patient identification information is used in RU-GASP.

### Antimicrobial susceptibility testing

At the SRCDV, the minimum inhibitory concentration (MIC, mg/L) of ceftriaxone (0.002-4 mg/L), spectinomycin (0.125-512 mg/L), azithromycin (0.002-4 mg/L), penicillin G (0.016-16 mg/L), and ciprofloxacin (0.002-128 mg/L) was determined using agar dilution method, according to the recommendations from the US Clinical and Laboratory Standards Institute [CLSI; 32]. All antimicrobial powder was purchased from Fluka Analytical (Steinheim, Germany). For azithromycin, for which the CLSI does not state any breakpoints, the MIC breakpoints from the European Committee on Antimicrobial Susceptibility Testing (EUCAST; http://www.eucast.org/clinical_breakpoints) were used. For quality control, as recommended by the CLSI [32] the *N. gonorrhoeae* reference strain АТСС 49226 was included in each testing. The 2008 WHO *N. gonorrhoeae* reference strains [26] were also included in the quality control on a regular basis. β-lactamase production was identified using nitrocefin discs, according to the manufacturer’s instructions (Cefinase discs; Becton Dickinson, Cockeysville, Md, USA).

### Isolation of genomic DNA

DNA was isolated from bacterial suspensions using the DNA express kit (Lytech Ltd, Moscow, Russia), according to the instructions from the manufacturer.

### Molecular epidemiological typing

For molecular epidemiological typing, NG-MAST was performed on gonococcal isolates from 2011 (n = 421) and 2012 (n = 100), as previously described [33]. NG-MAST allele numbers of the more variable segments of *porB* and *tbpB*, and sequence types (STs) were assigned using the NG-MAST website (http://www.ng-mast.net).

### Statistical analysis

Statistical analysis was performed using the Statistica software version 9.0 PL (StatSoft Corporation, Cracow, Poland). Z-test for comparison of proportions was used. The level of significance was set at *P* < 0.05.

## Results

### Patient characteristics

*N. gonorrhoeae* isolates (one isolate per patient) from 1200 patients (959 males and 241 females), 407 patients (324 males and 83 females), 423 patients (295 males and 128 females), and 106 (65 males and 41 females) in 2009, 2010, 2011 and 2012, respectively, were examined. The mean ages of the males (n = 1643) were 26.8 years (median age: 25 years; range: 15 to 64 years) and the mean ages of the females (n = 493) were 25.3 years (median age: 24 years; range: 16 to 76 years). The age distribution was relatively similar during the four years investigated.

### Antimicrobial susceptibility of *N. gonorrhoeae* isolates in 2009–2012 (n = 2136) in Russia

The results of the antimicrobial susceptibility testing of all isolates are summarized in Table 1.

**Table 1:** **Antimicrobial resistance and β-lactamase production in *Neisseria gonorrhoeae *** **isolates (n = 2136) from Russia in 2009–2012**

	**Number (%) of resistant isolates**			
	**2009 (n = 1200)**	**2010 (n = 407)**	**2011 (n = 423)**	**2012 (n = 106)**
**Ciprofloxacin**	533 (44.4)	217 (53.2)	138 (32.6)	27 (25.5)
(R > 0.5 mg/L)^*a*^				
**Penicillin G**	115 (9.6)	51 (12.5)	56 (13.2)	12 (11.3)
(R > 1 mg/L)^*a*^				
**Azithromycin**	28 (2.3)	20 (4.9)	70 (16.5)	18 (17.0)
(R > 0.5 mg/L)^*a*^				
**Spectinomycin**	16 (1.3)	18 (4.4)	49 (11.6)	1 (0.9)
(R > 64 mg/L)^*a*^				
**Ceftriaxone**	0	0	0	0
(R > 0.25 mg/L)^*a*^				
**β-lactamase production**	4 (0.3)	0	2 (0.5)	0

^*a*^Breakpoints for resistance according to the US Clinical and Laboratory Standards Institute [CLSI; 32].

For azithromycin, for which CLSI does not state any breakpoints, the resistance breakpoint from the European Committee on Antimicrobial Susceptibility Testing [EUCAST; http://www.eucast.org/clinical_breakpoints] was used.

**Figure 1:**
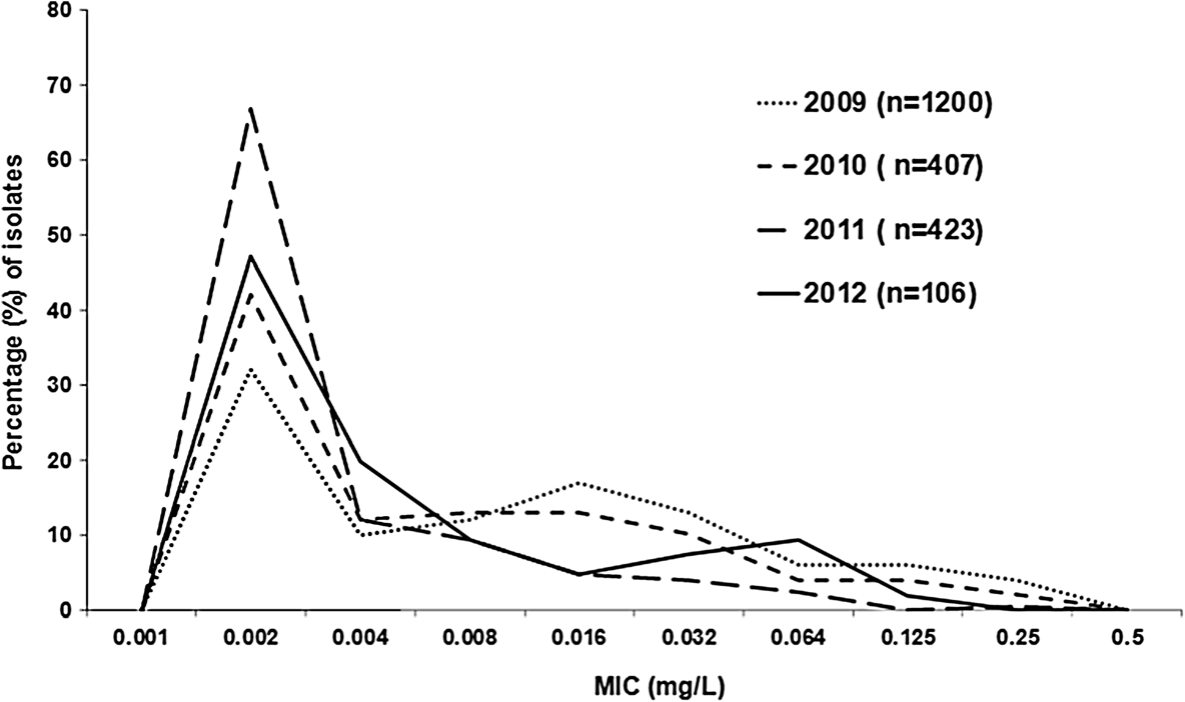
**The distribution of ceftriaxone MICs for *Neisseria gonorrhoeae *** **isolates (n = 2136) cultured in Russia from 2009 to 2012.**

Briefly, in 2012 the proportion of isolates with *in vitro* resistance was 25.5%, 17.0%, 11.3%, 0.9%, and 0% for ciprofloxacin, azithromycin, penicillin G, spectinomycin, and ceftriaxone, respectively. During 2009–2012, the proportions of *N. gonorrhoeae* isolates resistant to ciprofloxacin, penicillin G, azithromycin and spectinomycin ranged from 25.5% to 44.4%, 9.6% to 13.2%, 2.3% to 17.0% and 0.9% to 11.6%, respectively. The overall number of β-lactamase producing *N. gonorrhoeae* isolates was 6 (0.3%). In general, the resistance level to penicillin G was stable, the resistance level to ciprofloxacin was declining, however, the level of resistance to azithromycin was increasing significantly (*P* < 0.05) (Table 1). However, the highest MIC of azithromycin detected was 8 mg/L and no isolates with high-level resistance to azithromycin (MIC ≥ 256 mg/L) have yet been found in Russia. Worryingly, gonococcal isolates with low-level resistance to spectinomycin were identified in all the surveyed years. Nevertheless, no isolates with high-level resistance to spectinomycin (MIC ≥ 1024 mg/L) have yet been identified in Russia and the spectinomycin MICs of the identified isolates only ranged from 128 to 256 mg/L. All isolates from 2009 to 2012 were susceptible to ceftriaxone (Table 1). Nevertheless, using the European EUCAST breakpoint (http://www.eucast.org; R > 0.125 mg/L), in total 58 (2.7%) of the isolates during 2009–2012 were resistant to ceftriaxone. Interestingly, the prevalence of the isolates resistant to ceftriaxone according to the EUCAST breakpoint decreased significantly (*P* < 0.05), i.e. 48 (4.0%), 8 (2.0%), 2 (0.5%) and 0 (0%) isolates were found in 2009, 2010, 2011 and 2012, respectively. Furthermore, in general the annual MIC distribution for ceftriaxone appeared to shift to lower MICs during the study period 2009–2012 (Figure 1).

### *Neisseria gonorrhoeae* multiantigen sequence typing (NG-MAST)

The examined gonococcal isolates from 2011 (n = 421) and 2012 (n = 100) were assigned to 183 NG-MAST STs. Hundred twenty-two (66.7%) of these STs were not previously described. The most prevalent STs were ST807 (n = 41, 7.9% of isolates), ST5714 (n = 32, 6.1%), ST228 (n = 14, 2.7%), ST5042 (n = 11, 2.1%), ST1152 (n = 10, 1.9%), ST5825 (n = 10, 1.9%), ST5937 (n = 10, 1.9%), and ST5718 (n = 9, 1.9%). Five STs were represented by eight isolates, two STs by seven isolates, eight STs by six isolates, seven STs by five isolates, eight STs by four isolates, 18 STs by three isolates, 33 STs two isolates and remaining 98 STs were represented by single isolates.

In general, the most prevalent STs such as ST807, ST5714, ST228, ST5042, ST1152, ST5825, ST5937, and ST5718 had relatively low MICs of ceftriaxone, ranging from ≤0.016 mg/L to 0.064 mg/L. Notable, the two gonococcal isolates obtained in 2011 with a ceftriaxone MIC of 0.25 mg/L (resistant according to the European EUCAST breakpoint) were assigned as ST2861 and ST5929. One isolate assigned as ST1407, which is an internationally spread multidrug resistant gonococcal clone [8,12,15,19,20,34,35], was also identified in 2012 (in Ryazan, Central Federal District). This isolate was resistant to ciprofloxacin and, with exception of ceftriaxone, had similar antimicrobial resistance profile as ST1407 isolates described internationally. Surprisingly, the MIC of ceftriaxone was only 0.008 mg/L. Furthermore, the spectinomycin resistant isolates in 2011 and 2012 (n = 50) belonged to 32 different STs and were isolated in four of the seven Federal Districts of Russia. The most prevalent STs among the spectinomycin resistant isolates were ST5714 (n = 5), ST807 (n = 4), ST21 (n = 3), and ST5825 (n = 3).

## Discussion

The present study describes the antimicrobial resistance in *N. gonorrhoeae* isolates cultured from 2009 to 2012, and molecular epidemiological characteristics (NG-MAST) of *N. gonorrhoeae* isolates, obtained in 2011–2012, in Russia. Previously, only two minor NG-MAST studies examining Russian gonococcal isolates have been performed. Both these studies examined isolates cultured in 2004–2005 [29,30] and, accordingly, no knowledge of the NG-MAST STs of gonococcal strains currently circulating in Russia is available.

High prevalences of resistance to ciprofloxacin and penicillin G were observed. Similar high levels of resistance to these antimicrobials have been described from basically worldwide [3,6-12,21]. Accordingly, ciprofloxacin and penicillin G should not be recommended for empiric first-line antimicrobial monotherapy of gonorrhoea in Russia or in most other countries worldwide. Nevertheless, interestingly β-lactamase producing *N. gonorrhoeae* strains have remained rare in Russia [24,25,29] as well as in other independent countries of the former Soviet Union, e.g. Belarus [36]. This may indicate that penicillins have not been widely used for treatment of gonorrhoea in many years and/or that no imported β-lactamase producing gonococcal strains have been established and resulted in an endemic spread in Russia during several years. The prevalence of resistance to azithromycin was also high, particularly during the latest years, that is, 16-17% in 2011–2012. However, no isolates with high-level resistance to azithromycin (MIC ≥ 256 mg/L), which have been described from several other countries [37-42], have yet been identified in Russia. Worryingly, gonococcal isolates with resistance to spectinomycin, which are exceedingly rare internationally [3,6-9,12], were identified in all the surveyed years and in four of the seven Federal Districts of Russia. In earlier Russian studies [24,25], spectinomycin resistant gonococcal isolates have also been found in all the seven Federal Districts of Russia. In the present study, the spectinomycin resistant isolates (n = 50) belonged to 32 different STs. Accordingly, they did not represent any clonal spread and spectinomycin resistance has been selected in many different gonococcal strains. Spectinomycin remains also available and used for treatment of gonorrhoea in Russia, that is, despite that the level of use has decreased substantially during the last two decades. Fortunately, the resistant isolates had a spectinomycin MIC of maximum 128–256 mg/L and no isolates with high-level resistance to spectinomycin (MIC ≥ 1024 mg/L) [43-45] have yet been found in Russia. The molecular mechanisms for this low-level resistance to spectinomycin are commonly specific amino acid alterations in the ribosomal protein S5 [45,46], which has been selected by frequent use of spectinomycin. Using the CLSI breakpoints [32], all isolates from 2009 to 2012 were susceptible to ceftriaxone (MIC ≤ 0.25 mg/L). However, using the European EUCAST breakpoint (http://www.eucast.org), in total 58 (2.7%) of the isolates during 2009–2012 were resistant to ceftriaxone (MIC > 0.125 mg/L). Interestingly, the prevalence of these ceftriaxone resistant isolates decreased significantly (*P* < 0.05), i.e. from 4.0% in 2009 to 0% in 2012. Still no treatment failure of gonorrhoea with ceftriaxone has been verified in Russia, however, isolates with a ceftriaxone MIC of ≤0.25 mg/L have resulted in failures to treat pharyngeal gonorrhoea with ceftriaxone in other countries [13-15,17,18]. Similar increases in the susceptibility to extended-spectrum cephalosporins such as cefixime and ceftriaxone have recently been reported from the United Kingdom [47], Slovenia [48] and India [49]. The reasons for this remain unknown, however, this might indicate that mostly appropriate treatment with ceftriaxone (in adequately high dose and quality) with or without additional azithromycin (in dual therapy regimens) are used for treatment of gonorrhoea internationally. Accordingly, the use of less potent antimicrobials for treatment might have decreased. It is essential to continuously monitor, using MIC determination, the spread of gonococcal strains with multidrug resistance and resistance to particularly ceftriaxone, spectinomycin and azithromycin and, ideally, also the antimicrobial use/misuse in Russia as well as internationally. Most worryingly, the number of isolates examined in the RU-GASP has substantially decreased the latest years, which is due to both the increased use of genetic detection of *N. gonorrhoeae* for diagnosis of gonorrhoea as well as lack of sufficient financial and political commitments. A national surveillance, including representative gonococcal isolates from all the seven Russian Federal Districts, of gonococcal AMR (ideally also treatment failures) is imperative in Russia. Essential actions aiming to implement the recently published international action/response plans [21,22], and strengthen the culture capacity and surveillance of AMR and test-of-cure in Russia have been initiated. It is also crucial to establish and quality assure regional and national GASPs in the additional independent countries of the former Soviet Union and, for this purpose, national and international support, including political and financial commitment, is essential [3-5].

In Russia, for first-line empiric treatment of uncomplicated urogenital or extragenital gonorrhoea ceftriaxone (250 mg, intramuscularly), cefixime (400 mg, orally) or spectinomycin (2 g, intramuscularly) is recommended [27]. In practice, also fluoroquinolones, azithromycin, and other cephalosporins can be used in the treatment, and antimicrobials are easily available “over-the-counter”, which needs to be abandoned. Based on the present RU-GASP data and resistance emergence and spread worldwide [3,6-20,24,25,47,48], ceftriaxone should be the only option for first-line empiric antimicrobial monotherapy of gonorrhoea and it should be considered to increase the dose to 500 mg and/or add azithromycin (1–2 g) in the recommended first-line treatment, which is in line with the US CDC [50] and European [51] treatment guidelines. Furthermore, spectinomycin should be the alternative treatment option and only used when ceftriaxone is not available or the patient suffers from a severe β-lactam allergy. However, if pharyngeal gonorrhoea has not been excluded azithromycin is recommended to be added to the spectinomycin regimen. Cefixime, which is less potent compared to ceftriaxone and for which no data exist in Russia, should be excluded from the recommended first-line empiric treatment. This antimicrobial should only be used when injection therapy is refused by patient and should then ideally be used together with azithromycin, which is in line with the recently revised US CDC [50] and European [51] treatment guidelines. The dual antimicrobial regimens will also effectively eradicate any concomitant *Chlamydia trachomatis* infection.

NG-MAST analysis showed a diversified population of *N. gonorrhoeae* in Russia during 2011–2012, with 183 different NG-MAST STs identified among the examined 521 isolates. The high number of unique STs (n = 98) and STs that have not been previously described (n = 122) may be associated with the low number of cultured gonococcal isolates from each surveillance site, suboptimal diagnostics (only random gonorrhoea patients and/or isolates are identified), contact tracing (sexual contacts are not traced) and epidemiological surveillance (sexual transmission chains spreading an identical ST are not identified or followed-up), STs evolved locally in Russia (STs are not previously described because only two minor NG-MAST studies examining isolates from 2004–2005 [29,30] have been previously performed in Russia) or imported from abroad. Nevertheless, some main ST clusters of, e.g., ST807 (n = 41, 7.9% of isolates), ST5714 (n = 30), ST228 (n = 14), ST5042 (n = 11), ST1152 (n = 10), ST5825 (n = 10), and ST5937 (n = 10) were identified, which indicate some larger sexual transmission chains.

## Conclusions

In Russia, during 2009–2012 the diversified gonococcal population showed a high resistance to ciprofloxacin, penicillin G and azithromycin. Isolates with low-level resistance to spectinomycin were also identified each year. In general, the MICs of ceftriaxone were relatively high, however, they were decreasing significantly (*P* < 0.05) from 2009 to 2012. Ceftriaxone should be the only recommended first-line antimicrobial for empiric monotherapy of gonorrhoea in Russia. It should also be considered to increase the dose of ceftriaxone to 500 mg and/or add azithromycin (1–2 g) in the recommended first-line treatment, that is, use a dual antimicrobial therapy regimen [50,51]. Spectinomycin should be the second-line and only used when ceftriaxone is not available or the patient suffers from a severe β-lactam allergy. Regular, quality assured national and international surveillance of AMR (ideally also treatment failures) in *N. gonorrhoeae* is crucial and it is essential to further strengthen the RU-GASP in Russia.

## Competing interests

The authors declare that they have no competing interests.

## Authors’ contributions

AK, AK, NF, and MU designed and initiated the study. NF, RK, DV, VSe, and VSo coordinated and performed all the laboratory analyses. AK, NF and MU analysed and interpreted all the data, and wrote a first draft of the paper. All authors read, commented on and approved the final manuscript.

## Pre-publication history

The pre-publication history for this paper can be accessed here:

http://www.biomedcentral.com/1471-2334/14/342/prepub
